# Association of Ambulatory Ability on Complications and Medical Costs in Patients with Traumatic Spinal Cord Injury: A Decision-analytic Model

**DOI:** 10.7759/cureus.5337

**Published:** 2019-08-07

**Authors:** Larry E Miller, Louise H Anderson

**Affiliations:** 1 Clinical Research, Miller Scientific Consulting, Asheville, USA; 2 Health Economics, Technomics Research, Minneapolis, USA

**Keywords:** ambulation, function, hospitalization, paraplegia, pressure sore, sci, urinary tract infection

## Abstract

Purpose

The objective of this study was to determine the independent association of ambulatory ability with complications and medical costs in patients with spinal cord injury (SCI).

Methods

Patients with SCI between T1-T12 enrolled in the National Spinal Cord Injury Database (NSCID) provided a minimum one-year follow-up. Covariate-adjusted annual rates of important medical complications (pressure sore, urinary tract infection, hospitalization) and associated medical costs were determined over five years post-injury.

Results

A total of 1,753 patients provided data at one-year follow-up and 1,340 patients provided five-year data. At one-year post-injury, 82% of patients were non-ambulatory and 18% were ambulatory. After adjusting for important covariates, ambulatory status was associated with a lower annual probability of urinary tract infection (43% vs. 68%), pressure sore (12% vs. 35%), and hospitalization (23% vs. 34%). Covariate-adjusted base-case medical costs due to urinary tract infection, pressure sore, and hospitalization were 34% lower in ambulatory vs. non-ambulatory patients ($31,358 vs. $47,266) over five years. Probabilistic sensitivity analyses confirmed the base-case results.

Conclusion

In spinal cord-injured individuals, the ability to ambulate is independently associated with lower complication risks and associated medical costs over the five-year period following injury. Long-term clinical benefit and cost savings may be realized with assisted or unassisted ambulation in spinal cord-injured patients.

## Introduction

Recovery of ambulation is one of the main goals of patients following spinal cord injury (SCI) [1]. The inability to ambulate has profound negative consequences on the physical health and quality of life of individuals with SCI. Sedentary lifestyle predisposes spinal cord-injured individuals to medical complications such as cardiovascular disease, osteoporosis, and pressure ulcers at a higher frequency than that of the general population [2-3]. Further, approximately 30% of spinal cord-injured adults are admitted to the hospital each year, with an average of 22 hospital days annually [4]. The resulting economic impact of SCI-related complications accounts for more than $3 million over the lifespan of a patient [5]. 

Given that routine physical activity lowers the risk for chronic disease in the general population, comparable risk reductions in spinal cord-injured adults may be a reasonable expectation. Intermittent standing and habitual ambulation improve upper body muscular fitness, slows the decline in bone mineral density by exposure to gravitational and muscular loading forces, improves circulatory responses, and reverses a number of the health risks associated with prolonged sitting [6-9]. Further, those who transition from wheelchair use at rehabilitation discharge to walking at one year report the highest quality of life among spinal cord-injured patients [10]. Yet the typical spinal cord-injured patient engages in only 40% of the activity levels of able-bodied peers, which is insufficient to yield meaningful health benefits [11]. Many patients who may benefit from increased physical activity face barriers to participation such as lack of assistive equipment, transportation, and/or access to accommodating facilities. Furthermore, patients and caregivers may be hesitant to participate in such programs due to safety concerns related to falling or exacerbation of spasticity and contractures. If issues related to patient access and safety could be overcome, it is plausible that the risk of SCI-related complications and associated medical costs could be partially offset by routinely engaging in modest volumes of assisted or unassisted ambulation. We hypothesized that greater ambulatory ability would be associated with a lower risk of complications and associated medical costs in spinal cord-injured adults.

## Materials and methods

Model structure

**Figure 1:**
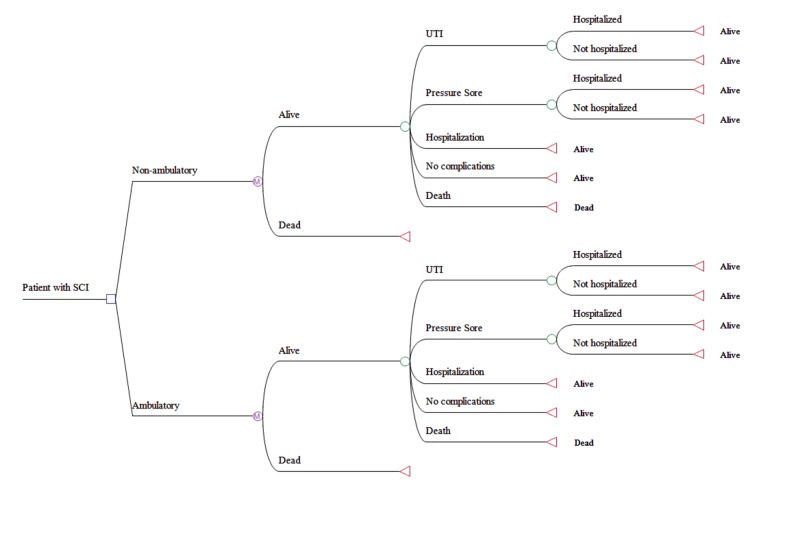
Markov model structure SCI, spinal cord injury; UTI, urinary tract infection

A Markov model was developed to estimate complications and medical costs in SCI patients over a five-year period following discharge from inpatient rehabilitation. Ambulatory and non-ambulatory groups were modeled; the groups had similar Markov processes. Patients entered the model following discharge from an acute care treatment facility. In each cycle, patients could experience one of five distinct outcomes: a) urinary tract infection (UTI), with or without hospitalization, b) pressure sore, with or without hospitalization, c) hospitalization for any reason other than UTI or pressure sore, d) no complications, or e) death (Figure 1).

Complication data sources

Data were obtained from the Spinal Cord Injury Model Systems (SCIMS), a network of federally funded facilities that collects data on demographic and clinical characteristics on approximately 13% of spinal-cord individuals in the United States [12]. Data were extracted from the National Spinal Cord Injury Database (NSCID). The NSCID contains data from individuals who a) sustained an SCI due to a traumatic event, b) reside in geographical proximity to a SCIMS center at the time of injury, c) were admitted to a SCIMS center within one year of injury, d) were discharged from the SMISC center as either neurologically normal, completed rehabilitation, or deceased, and e) provided informed consent. For this study, eligible patients were adults who experienced traumatic SCI between 2000 and 2014, presented with a level of injury between T1 and T12, and provided minimum one-year follow-up data. Institutional review board approval was obtained at the National Spinal Cord Injury Statistical Center and an informed consent waiver for this research was granted by IntegReview Institutional Review Board (Austin, TX).

Patient characteristics assessed at the time of rehabilitation discharge included age, sex, and American Spinal Injury Association (ASIA) Impairment Scale. The characteristics measured at one- and five-year follow-up were age, marital status, body mass index (BMI), education status, working status, complications, primary insurance, and functional independence measure (FIM) mobility score. Patients self-reported medical complications over the preceding year at one and five years. Clinical complications included UTI requiring antibiotic treatment, pressure sore with open/broken skin, and overnight hospitalization for any reason. UTI and pressure sore were reported as yes/no; multiple occurrences of the same complication within the preceding year could not be evaluated. Hospitalization was reported as yes/no for any reason within the preceding year and the number of inpatient admissions. Additional information about outcomes and definitions is provided in the NSCID Data Dictionary [13].

Treatment cost data sources

Standard outpatient treatments for UTI and pressure sore complications were determined following consultation with a urologist regarding UTI treatment and a wound care specialist and occupational therapist regarding pressure sore treatment. Outpatient treatment costs were estimated from Medicare national fee schedules. To estimate cost per inpatient stay, Medicare inpatient claims with diagnosis codes for SCI (ICD-9 806.xx) were analyzed. For this study, hospitalizations occurred after discharge from inpatient rehabilitation. Therefore, the claims analysis excluded index SCI claims, which were identified by a primary diagnosis of SCI (806.xx) or an accident code (E000.x-E999.x) in any position. Data were obtained from the 2014 Medicare Inpatient Hospital Standard Analytic Files. The average cost per inpatient stay was adjusted to 2016 using the medical care services Consumer Price Index.

Data analysis

Patients were classified as ambulatory or non-ambulatory based on the self-reported ability to walk 150 feet, walk one block, and climb one flight of stairs. Patients who could perform all the activities were classified as ambulatory; those who could perform none of the activities were classified as non-ambulatory. Patients who could complete some, but not all, activities were not included in the analysis.

Demographic and complication descriptive data were reported for each ambulation group at one- and five-year follow-up. Odds ratios (OR) and 95% confidence intervals derived from logistic regression analysis were used to estimate the independent association of ambulation on complication rates after adjusting for age, sex, marital status, employment status, ASIA score, and injury level. Logistic model parameter estimates and covariate mean values were used to estimate the annual probability of each outcome. Life table methods were used to estimate annual mortality, which was converted to monthly probabilities for the Markov model. Data analyses were performed using SAS version 9.3 (SAS Institute, Inc., Cary, NC).

Cohort Markov modeling analysis was performed using TreeAge Pro 2016 (TreeAge Software, Inc., Williamstown, MA). The model had a five-year time horizon with monthly cycles. Event probabilities for years 2, 3, and 4 were calculated by linear interpolation from years 1 to 5. Medical costs were in 2016 US dollars and discounted at 3% per year; a payer perspective was taken. One-variable sensitivity analysis was evaluated using the 95% confidence interval bounds for each variable. Probability distributions were created for the variables and used in probabilistic sensitivity analyses (PSA), during which 1,000 simulations were performed.

## Results

Patient characteristics

A total of 1,753 patients (82% non-ambulatory, 18% ambulatory) provided data at one-year follow-up. Non-ambulatory patients were younger (*p *<0.01) and less likely to be married (*p *= 0.01) or employed (*p *<0.01). Non-ambulatory patients presented with a higher level of injury (*p *<0.001), greater impairment on the AIS (*p *<0.01), and lower FIM mobility total score (*p *<0.001) relative to ambulatory patients. Urinary tract infection (76% vs. 34%), pressure sore (40% vs. 5%), and hospitalization for any reason (37% vs. 19%) were more frequently reported in non-ambulatory vs. ambulatory patients at one-year follow-up (all *p *<0.001; Table 1). For hospitalized patients, the average number of inpatient stays was 1.7 for non-ambulatory and 1.4 for ambulatory patients. Similar differences in patient characteristics were observed between groups in the 1,340 patients who provided data at five-year follow-up. Higher complication rates in non-ambulatory patients were also observed at five-year follow-up (Appendix Table 6).

**Table 1:** Patient characteristics at one-year post-injury ASIA, American Spinal Injury Association; FIM, functional independence measure; SD, standard deviation

Variable	Non-ambulatory	Ambulatory	P-value
Number of Participants	1,440	313	
Demographics			
Age, Mean (SD)	35.6 (14.4)	39.1 (17.5)	<0.01
Male, % (n/N)	80.0 (1152/1440)	75.4 (236/313)	0.07
Married, % (n/N)	31.8 (457/1439)	38.9 (121/311)	0.01
Body mass index, Mean (SD)	25.5 (6.5)	26.7 (6.7)	0.7
High school education, or equivalent, % (n/N)	55.3 (793/1334)	54.5 (170/312)	0.8
Working, % (n/N)	12.4 (178/1133)	31.7 (99/312)	<0.01
ASIA Impairment Scale, % (n/N)			<0.01
A	76.6 (1102/1440)	6.4 (20/313)	
B	11.7 (169/1440)	2.6 (8/313)	
C	9.9 (142/1440)	22.1 (69/313)	
D	1.7 (25/1440)	68.9 (215/313)	
Unknown	0.1 (2/1440)	0.3 (1/313)	
Level of injury, % (n/N)			<0.001
T1-T6	48.1 (693/1440)	32.3 (101/313)	
T7-T12	51.9 (747/1440)	67.7 (212/313)	
Complications, % (n/N)			
Urinary tract infection	75.7 (412/544)	34.1 (47/138)	<0.001
With genitourinary hospitalization	16.8 (69/412)	10.6 (5/47)	0.3
Pressure sore	39.6 (215/543)	5.1 (7/138)	<0.001
With pressure sore/disease of skin hospitalization	19.1 (41/215)	0.0 (0/7)	0.2
Hospitalization, all cause % (n/N)	36.8 (526/1429)	18.8 (58/309)	<0.001
Number of in-patient stays Mean, (SD)	1.7 (1.2)	1.4 (0.7)	0.02
Number of days hospitalized Mean, (SD)	16.7 (30.3)	12.3 (26.4)	0.03
FIM Mobility Total Score, Mean (SD)	69.3 (14.2)	85.6 (5.8)	<0.001

Complication rates

After adjusting for covariates, greater ambulatory ability was associated with a significant reduction in complication rates. The odds of UTI for the ambulatory group was considerably lower than that of the non-ambulatory group (year 1 OR=0.454, p<0.01; year 5 OR=0.256, p<0.01) (Appendix Table 7). The odds of pressure sore for the ambulatory group was approximately one-fourth that of the non-ambulatory group (year 1 OR=0.235, p<0.01; year 5 OR=0.264, p<0.01) (Appendix Table 8). The odds of all-cause hospitalization for the ambulatory group were also significantly reduced during follow-up relative to the non-ambulatory group (year 1 OR=0.585, p=0.03; year 5 OR=0.567, p=0.04) (Appendix Table 9).

Treatment costs

Payer cost of an inpatient stay for a spinal cord-injured patient was estimated at $17,985. This estimate was based on 1,383 inpatient stays for 1,214 patients (mean age: 74 years, 54% males) during 2014. The mean length of stay was 10.9 (SD: 10.9) days per admission. Treatment cost was estimated at $229 for outpatient UTI treatment and $1,803 for outpatient pressure sore treatment (Appendix Table 10).

Covariate-adjusted base case for decision-analytic model

Covariate-adjusted base case model inputs are shown in Table 2. Simulated patients in the ambulatory group had lower probabilities of UTI, pressure sore, and all-cause hospitalization. The probability of death, number of inpatient stays for patients with hospitalization, and treatment costs did not vary by ambulatory status. Over five years, medical costs due to UTI, pressure sore, and hospitalization were 34% lower in ambulatory vs. non-ambulatory patients ($31,358 vs. $47,266). The number of persons with UTI was 37% lower, number with pressure sore was 66% lower, and the number with all-cause hospitalization was 33% lower in ambulatory vs. non-ambulatory groups. The annual probabilities of UTI (43% vs. 68%), pressure sore (12% vs. 35%), and hospitalization (23% vs. 34%) were lower in ambulatory patients (Table 3).

**Table 2:** Base case model variable values

Annual Probabilities	Projection Year	Non-ambulatory	Ambulatory
Urinary tract infection	1	73.3%	55.6%
	5	62.4%	29.8%
Pressure sore	1	33.1%	10.4%
	5	37.7%	13.8%
Hospitalization, all cause	1	34.7%	23.8%
	5	34.1%	22.7%
Death	All years	1.5%	1.5%
Number of inpatient stays	All years	1.6	1.6
Costs			
Urinary tract infection treatment	$229		
Pressure sore treatment	$1,803		
Inpatient stay	$17,985		
Discount rate	3%		

 

**Table 3:** Covariate-adjusted base case five-year results Hospitalized persons were assumed to have 1.6 inpatient stays per year.

Variable	Non-ambulatory	Ambulatory
Population size	10,000	10,000
Average healthcare cost	$47,266	$31,358
Number of persons		
Urinary tract infection, all	32,715	20,635
Urinary tract infection, hospitalized	5,236	1,540
Pressure sore, all	17,021	5,814
Pressure sore, hospitalized	3,387	0
Hospitalized, other causes	7,948	9,644
Death	746	746
Annual probability, 5-year average		
Urinary tract infection	68.0%	42.9%
Pressure sore	35.4%	12.1%
Hospitalization, all-cause	34.4%	23.2%
Death	1.6%	1.6%

Covariate-adjusted sensitivity analysis for decision-analytic model

**Figure 2:**
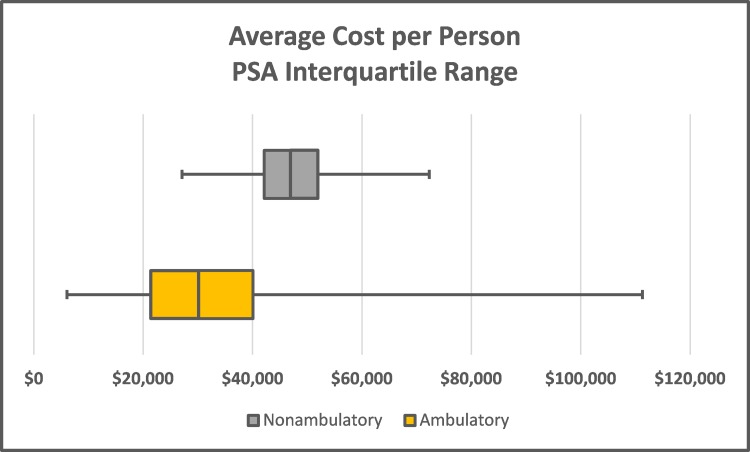
Boxplots demonstrating cost per person by ambulatory group in probabilistic sensitivity analysis PSA, probabilistic sensitivity analysis

**Figure 3:**
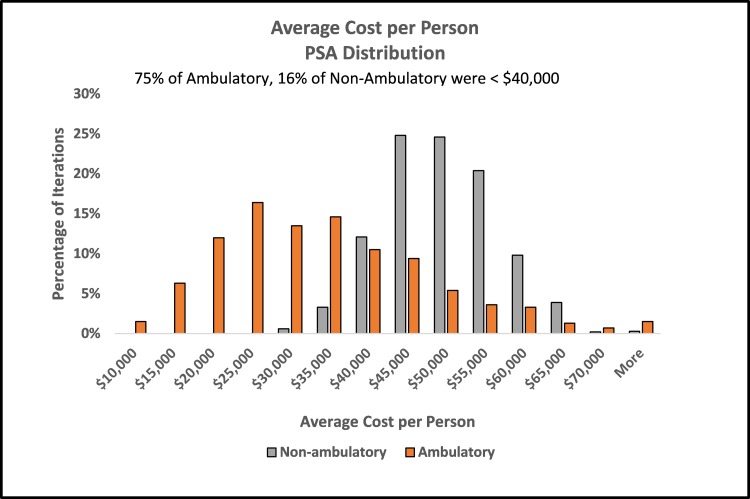
Distribution of cost per person by ambulatory group in probabilistic sensitivity analysis PSA, probabilistic sensitivity analysis

**Figure 4:**
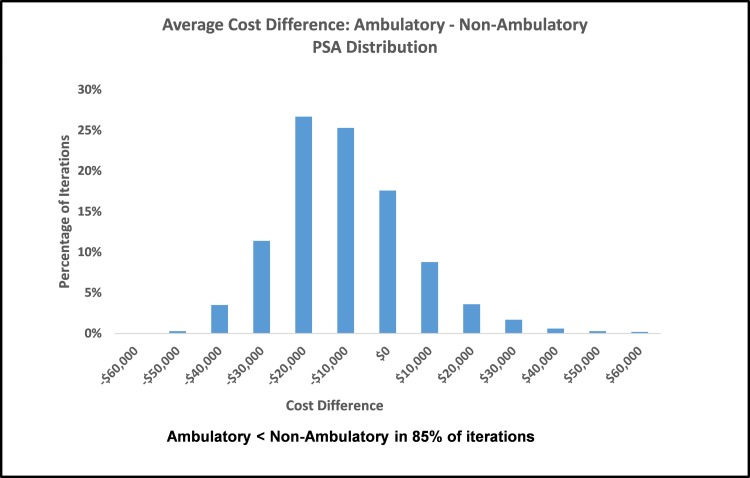
Distribution of cost difference per person comparing ambulatory to non-ambulatory groups in probabilistic sensitivity analysis PSA, probabilistic sensitivity analysis

One-variable sensitivity analysis was used to test the influence of variability in complication rates and cost of inpatient stays on average costs. Each variable was tested at its upper and lower 95% confidence interval bounds (Appendix Table 11). The most influential variable in the model was hospitalization rate. In the non-ambulatory group, average cost per person was 26% lower at the lowest hospitalized rate and 35% higher at the highest rate compared to the base case result. Other complication rates and cost of inpatient stays impacted the average overall cost by no more 5% in either direction compared to base-case results (Appendix Figure 5). Due to the smaller sample size, the 95% confidence intervals were wider in the ambulatory group. In these patients, hospitalization rate also had the greatest influence, impacting the average cost per person from -54% to +124% compared to base-case results. Other complication rates and cost of inpatient stays impacted the ambulatory average cost by - 6% to +12% (Appendix Figure 6). Probabilistic sensitivity analysis allowed complication rates for UTI, pressure sore, and hospitalized and cost of inpatient stay to vary within their defined distributions. In PSA, the average cost per person remained lower in ambulatory vs. non-ambulatory patients ($31,915 vs. $47,113; Figure 2, Table 4). The distribution of PSA results showed that 75% of ambulatory group iterations had an average cost per person of $40,000 or less compared to 16% of the non-ambulatory group iterations (Figure 3). When comparing groups, complication costs were lower in ambulatory patients in 85% of simulations (Figure 4, Table 5).

**Table 4:** Distribution of cost per person in probabilistic sensitivity analysis

Value	Non-ambulatory	Ambulatory
Minimum	$27,087	$6,091
25^th^ Percentile	$42,123	$21,378
Median	$46,941	$30,149
Mean	$47,113	$31,915
75^th^ Percentile	$51,937	$40,065
Maximum	$72,292	$111,277

**Table 5:** Distribution of cost difference per person comparing ambulatory to non-ambulatory groups in probabilistic sensitivity analysis

Cost Difference	Frequency	Percentage	Cumulative
Less than -$60,000	0	0%	0%
-$60,000 to -$50,000	3	0%	0%
-$50,000 to -$40,000	35	4%	4%
-$40,000 to -$30,000	114	11%	15%
-$30,000 to -$20,000	267	27%	42%
-$20,000 to -$10,000	253	25%	67%
-$10,000 to $0,000	176	18%	85%
$0,000 to $10,000	88	9%	94%
$10,000 to $20,000	36	4%	97%
$20,000 to $30,000	17	2%	99%
$30,000 to $40,000	6	1%	100%
$40,000 to $50,000	3	0%	100%
More than $60,000	2	0%	100%

## Discussion

SCI is responsible for a significant lifelong loss of functional ability, frequent secondary complications, and tremendous costs to the healthcare system. We used covariate-adjusted complication probabilities within a decision-analytic model framework to estimate complications and associated medical costs in spinal cord-injured adults over a five-year period following discharge from inpatient rehabilitation. The major outcomes of this study were lower adjusted annual probabilities of UTI (43% vs. 68%), pressure sore (12% vs. 35%), and hospitalization (23% vs. 34%), with a 34% reduction in associated medical costs over five years in ambulatory SCI patients.

The results of this study are in agreement with conclusions drawn from other studies on the association of physical activity on complications and costs in spinal cord-injured patients. Cohen et al. reported that a higher FIM score at rehabilitation discharge was associated with fewer hospitalizations, fewer days hospitalized, and reduced need for assistive care over long-term follow-up [14]. DeJong et al. reported that more intensive physical therapy regimens during inpatient rehabilitation and regular exercise after discharge rehabilitation lowered the odds of rehospitalization in the first year following injury [15]. While these studies further illustrate the potential benefits of increased physical activity in spinal cord-injured patients, they fail to take into consideration the potential influence of variables such as level of injury, AIS score, and patient age. The current study extends this previous research by highlighting the independent association of ambulatory ability on complication risk and associated medical costs when controlling for these important confounders.

Within the framework of the current decision-analytic model, two important considerations are worth mention. The first consideration relates to the reimbursement assumptions inherent within the model. Hospital reimbursements for the same procedures differ widely among payers, with public payers (Medicare and Medicaid) setting a rate that is approximately 62% that of private payers [16]. In the first five years following SCI, Medicare/Medicaid are the primary payers in 40% to 55% of patients while private payers, Veteran’s Administration, and workman’s compensation comprise the remainder. Since Medicare reimbursements may be lower than that of other payers, our cost estimates may be similarly underestimated. A second consideration relates to additional costs attributable to SCI that were not included in this study. Rehospitalizations account for only 30% to 42% of the total annual costs attributable to care of spinal cord-injured patients while factors not measured in this study such as attendant care, equipment, supplies, medications, and environmental modifications account for the remainder of costs [5]. Therefore, our estimates of lower medical costs in ambulatory SCI patients may comprise only a fraction of the total cost savings attributable to ambulation. If one were to adjust costs based on typical reimbursement ratios among different payers and reasonably assume that unmeasured costs in the current study (e.g. due to attendant care, medications, equipment, environmental modifications) may be reduced to a similar degree as the medical costs reported in this study, then the total annual cost savings attributable to ambulation in spinal cord-injured adults may be several-fold greater than that reported here.

There were several strengths and limitations of this research that warrant discussion. Strengths of this research include a large sample size of spinal cord-injured adults, excellent generalizability due to geographic and patient diversity, and standardized data collection methods across NSCID centers. A number of limitations with this research were also identified. First, although outcomes were adjusted for influential covariates such as AIS and level of injury in order to account for group imbalances, unmeasured variables not accounted for in the statistical analysis may bias the results of this study. Second, the only complications and associated costs considered in this study were those reported in the NSCID, namely UTI, pressure sore, and hospitalization following discharge from inpatient rehabilitation. To the extent that spinal cord-injured patients may incur additional significant costs, this study does not provide a complete assessment of the potential overall cost savings due to greater ambulation in SCI patients. Third, complication reporting was dependent on patient recall over the previous year and is therefore prone to possible error or recall bias. Finally, the number of complications requiring outpatient care experienced during any interval was not assessed. However, the impact of this was likely minimal since UTI and pressure sore treatment accounted for a small portion of overall SCI-related treatment costs.

## Conclusions

In spinal cord-injured individuals with thoracic lesions, the ability to ambulate is independently associated with lower complication risks and associated medical costs over the five-year period following injury. Long-term clinical benefit and cost savings may be realized with assisted or unassisted ambulation in spinal cord-injured patients. The main limitations of this analysis include the potential for recall bias and the possibility that unmeasured variables not accounted for in the statistical analysis biased study results.

## Figures and Tables

**Figure 5:**
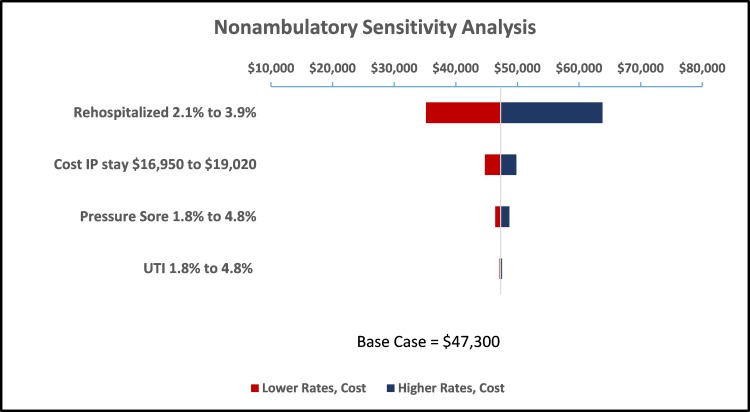
Sensitivity analysis of costs in non-ambulatory patients IP, in-patient; UTI, urinary tract infection.

**Figure 6:**
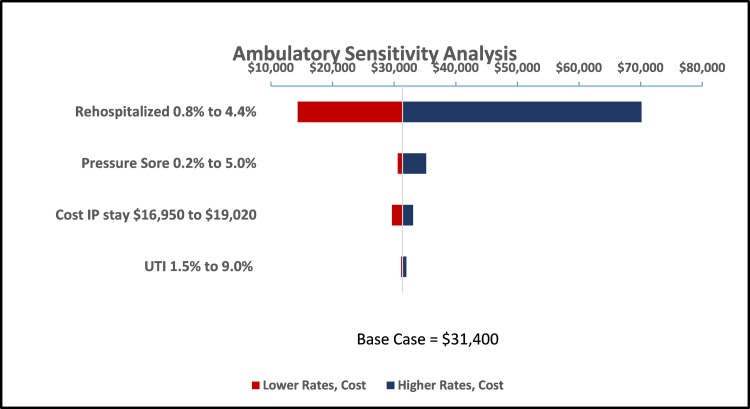
Sensitivity analysis of costs in ambulatory patients IP, in-patient; UTI, urinary tract infection.

## Appendices

**Table 6:** Patient characteristics at five years post-injury ASIA, American Spinal Injury Association; FIM, functional independence measure; GED, general education diploma; SD, standard deviation; UTI, urinary tract infection

Variable	Non-ambulatory	Ambulatory	P-value
Number of Participants	1,125	215	
Demographics			
Age, Mean (SD)	38.1 (13.6)	41.1 (5.8)	0.01
Male, % (n/N)	77.9 (876/1125)	74.9 (161/215)	0.3
Married, % (n/N)	31.5 (354/1125)	43.7 (94/215)	<0.001
BMI, Mean (SD)	27.9 (7.2)	26.8 (5.4)	0.06
High School or GED, % (n/N)	55.5 (621/1119)	51.6 (111/215)	0.3
Working, % (n/N)	20.6 (231/1120)	39.9 (85/213)	<0.001
ASIA Impairment Scale, % (n/N)			<0.001
A	79.9 (899/1125)	10.7 (23/215)	
B	11.7 (132/1125)	5.1 (11/215)	
C	6.8 (76/1125)	24.6 (53/215)	
D	1.3 (15/1125)	58.6 (126/215)	
Unknown	0.3 (3/1125)	0.9 (2/215)	
Level of injury, % (n/N)			<0.001
T1-T6	47.0 (529/1125)	28.4 (61/215)	
T7-T12	53.0 (596/1125)	71.6 (154/215)	
Complications, % (n/N)			
UTI	63.5 (294/463)	22.0 (20/91)	<0.001
UTI and Genitourinary hospitalization	15.0 (44/294)	0.0 (0/20)	0.06
Pressure sore	40.1 (187/466)	9.9 (9/91)	<0.001
Pressure sore and Disease of skin hospitalization	20.9 (39/187)	0.0 (0/9)	0.1
Hospitalization, % (n/N)	36.0 (403/1120)	18.8 (49/213)	<0.001
Number of in-patient stays Mean, (SD)	1.5 (1.0)	1.4 (0.7)	0.3
Number of days hospitalized Mean, (SD)	19.8 (34.9)	8.4 (13.5)	<0.001
FIM Mobility Total Score, Mean (SD)	71.4 (13.9)	86.5 (4.9)	<0.001

**Table 7:** Logistic regression results of covariate association on urinary tract infection risk ASIA, American Spinal Injury Association; UTI, urinary tract infection

Dependent Variable (0/1)	UTI Year 1			UTI Year 5		
N=0	217			240		
N=1	458			312		
Total N	675			552		
Independent Variables	Beta	Odds Ratio	p-value	Beta	Odds Ratio	p-value
Intercept	-0.645	0.525	0.164	-0.024	0.977	0.964
Ambulatory	-0.790	0.454	0.009	-1.364	0.256	0.000
Age	0.008	1.008	0.271	0.000	1.000	0.956
Female	0.567	1.763	0.023	0.522	1.686	0.025
Married	-0.027	0.974	0.907	-0.149	0.861	0.510
Work	-0.365	0.694	0.135	-0.009	0.991	0.968
ASIA A vs D	1.496	4.462		0.686	1.986	0.099
ASIA B vs D	1.067	2.907	0.013	0.471	1.601	0.311
ASIA C vs D	0.813	2.254	0.021	0.008	1.008	0.986
Injury level T1-T6	0.367	1.443	0.049	-0.154	0.858	0.414

**Table 8:** Logistic regression results of covariate association on pressure sore risk ASIA, American Spinal Injury Association

Dependent Variable (0/1)	Pressure Sore Year 1			Pressure Sore Year 5		
N=0	454			359		
N=1	222			194		
Total N	676			553		
Independent Variables	Beta	Odds Ratio	p-value	Beta	Odds Ratio	p-value
Intercept	-2.163	0.115	0.001	-0.024	0.255	0.964
Ambulatory	-1.448	0.235	0.004	-1.364	0.264	0.000
Age	0.022	1.022	0.002	0.000	1.017	0.956
Female	-0.129	0.879	0.582	0.522	0.712	0.025
Married	-0.669	0.512	0.004	-0.149	0.670	0.510
Work	-0.652	0.521	0.022	-0.009	0.526	0.968
ASIA A vs D	1.371	3.939	0.010	0.686	2.294	0.099
ASIA B vs D	1.163	3.199	0.044	0.471	1.219	0.311
ASIA C vs D	-0.291	0.748	0.629	0.008	1.514	0.986
Injury level T1-T6	0.104	1.109	0.568	-0.154	0.915	0.414

**Table 9:** Logistic regression results of covariate association on hospitalization risk ASIA, American Spinal Injury Association.

Dependent Variable (0/1)	Hospitalization Year 1			Hospitalization Year 5		
N = 0	1146			881		
N = 1	582			440		
Total N	1728			1321		
Independent Variables	Beta	Odds Ratio	p-value	Beta	Odds Ratio	p-value
Intercept	-1.820	0.162	0.000	-1.663	0.190	0.000
Ambulatory	-0.536	0.585	0.030	-0.567	0.567	0.043
Age	0.022	1.022	0.000	0.022	1.023	0.000
Female	0.210	1.234	0.101	0.081	1.084	0.576
Married	-0.245	0.783	0.057	-0.218	0.804	0.128
Work	-0.456	0.634	0.004	-0.620	0.538	0.000
ASIA A vs D	0.659	1.932	0.020	0.467	1.595	0.163
ASIA B vs D	0.244	1.277	0.440	0.115	1.122	0.756
ASIA C vs D	0.397	1.487	0.147	0.266	1.305	0.426
Injury level T1-T6	0.001	1.001	0.995	-0.038	0.963	0.754

**Table 10:** Derivation of complication treatment costs CPT, current procedural terminology; ICD, International Classification of Diseases; OT, occupational therapy; SCI, spinal cord injury; SD, standard deviation; UTI, urinary tract infection *Medication cost assumed 14 tablets; antibiotics Bactrim Supra, Cipro, and Macrobid were assumed to be prescribed equally; a $4.00 dispensing fee was added. **Wound clinic initial evaluation and treatment was an average of costs for stage 2, 3, and 4 wounds, size between 20 cm^2^ and 40cm^2^. Stage 2 office visit CPT 99213; stage 3 office visit and debridement CPTs 999203, 97597, 97598; stage 3 office visit and debridement CPTs 99204, 11042, 11045.

Inpatient stay Reimbursement		
SCI patients, ICD-9 diagnosis 806.xx		
Number	1,214	
Age, Mean (SD)	73.7 (12.0)	
Male, % (N)	54% (655)	
Length of stay per admission, Mean (SD)	10.9 (10.9)	
Inpatient stays		
Number	1,383	
Reimbursement, Mean (SD)	$17,985 (528)	
Urinary tract infection treatment reimbursement		
Type of service	Reimbursement	CPT codes
Physician office visit	$108.13	99214
Post-void residual test	$19.33	51798
Urinalysis	$5.84	81000
Urine culture, initial	$42.46	87086, 87088, 87184
Antibiotic medication*	$10.80	
Urine culture, repeat	$42.46	87086, 87088, 87184
Total UTI treatment	$229.02	
Pressure Sore treatment		
Type of service	Reimbursement	CPT codes
Physician office visit	$90.77	99213, 99214
Wound clinic, initial**		
Average Stage 2, 3, 4	$202.89	
Adequate blood flow test	$90.58	93922
Wound culture	$17.38	87071
Total Wound clinic, initial	$310.85	
Wound clinic follow-up, 12 wks	$1,207.32	97597, 97598
Occupational Therapy (OT)		
Evaluation	$85.57	97003
Wheelchair cushion (used by 50%)	$133.60	E2603
Wheelchair management (used by 33%)	$124.60	97542
Total OT	$193.90	
Total Pressure Sore treatment	$1,802.84	

**Table 11:** 95% confidence intervals of sensitivity analysis assumptions CI: confidence interval; UTI, urinary tract infection. Beta distribution was used for event probabilities, normal distribution for cost of inpatient stay.

	Non-ambulatory				Ambulatory			
Probabilities	Base		95% CI		Base		95% CI	
	Annual	Monthly	Lower	Upper	Annual	Monthly	Lower	Upper
UTI	68.3%	5.7%	4.0%	8.1%	45.3%	3.8%	1.5%	9.0%
Pressure Sore	35.2%	2.9%	1.8%	4.8%	11.8%	1.0%	0.2%	5.0%
Hospitalizations	34.5%	2.9%	2.1%	3.9%	23.3%	1.9%	0.8%	4.4%
			All patients, 95% confidence interval					
Cost			Base	Lower	Upper			
Inpatient stay			$17,985	$16,950	$19,020			
